# Therapeutic drug monitoring of daptomycin in a critically ill patient cohort

**DOI:** 10.3389/fmed.2026.1732645

**Published:** 2026-02-11

**Authors:** Rubi Stephani Hellwege, Mattia Müller, Rolf Erlebach, Onur Sazpinar, Alix Buhlmann, Reto A. Schuepbach, Sascha David, Daniel A. Hofmaenner

**Affiliations:** 1 Institute of Intensive Care Medicine, University Hospital Zurich, Zurich, Switzerland; 2 Department of Nephrology, Medical School Hannover, Hannover, Germany

**Keywords:** antibiotics, daptomycin, intensive care unit, side effects, trough level

## Abstract

**Background:**

Pharmacokinetics of antimicrobial agents are altered in critically ill patients. Therefore, therapeutic drug monitoring (TDM) has gained much attention in recent years. Specifically, the role of TDM for daptomycin, its potential influence on dose adaptations and subsequent daptomycin levels, as well as daptomycin-related side effects is so far unclear in the critical care setting.

**Methods:**

This was a retrospective study including critically ill, adult patients from January 2010 to July 2023. No criteria for TDM were predefined and the decision to perform TDM was left to the discretion of the treating physician. The primary outcome was the evaluation of baseline daptomycin trough levels in patients undergoing TDM, and how subsequent levels were affected by potential dose adaptations. Further outcomes included daptomycin-free days alive over 14 days and the occurrence of side effects between patients with and without daptomycin TDM.

**Results:**

Two hundred seventy patients were included. The patient group was heterogeneous regarding elective or emergency admissions and had surgical and medical underlying conditions. Over the ICU stay, median daptomycin trough levels were 9 mg/L (IQR 5.8–16.4 mg/L) and median peak levels were 29. 8 mg/L (IQR 14.8 – 46 mg/L). The first measured daptomycin trough level was too low (<10 mg/L) in 62 patients (54.4%) with TDM. Despite dosage increases in 14 patients (22.6%), median subsequent levels did not increase and were only 7.0 mg/L (IQR 4.8–13.6 mg/L). Patients who underwent TDM experienced significantly more frequent daptomycin dose increases than those who did not (28.8% vs. 13.1%, *p* = 0.002). Patients with TDM experienced fewer daptomycin-free days alive compared to patients without TDM [5 days (IQR 0–8 days) vs. 10 days (IQR 8–11 days), *p* < 0.001]. Increases from baseline in creatine kinase levels and eosinophil counts during daptomycin treatment did not differ significantly between patients with and without TDM.

**Conclusion:**

Daptomycin levels might be commonly low in critically ill patients and often appear not to increase after dose adjustments. TDM was associated with more frequent dose escalations and fewer daptomycin-free days, but did not significantly reduce the incidence of adverse events in a large cohort of critically ill patients.

## 1 Introduction

Daptomycin is a cyclic lipopeptide antibiotic used to treat severe Gram-positive bacterial infections (1, 2). Some studies have shown that pharmacokinetics of daptomycin can be significantly altered in critically ill patients, particularly in patients receiving extracorporeal support, where drug sequestration within the circuit might be a crucial factor (3, 4). In addition, renal replacement therapy (RRT) has been demonstrated to influence drug clearance, with the potential to result in drug under- or overexposure (3, 5–7). Further reasons for altered pharmacokinetics with the potential necessity for dosing adjustments are multifactorial and include altered resorption, distribution, increased volume of distribution, protein binding, liver impairment and various degrees of renal impairment/failure, augmented renal clearance, and other influences of extracorporeal devices (8–12).

Consequently, therapeutic drug monitoring (TDM) has been proposed as an effective measure to monitor serum levels of a wide range of antimicrobials to achieve sufficient therapeutic ranges and to minimize toxic effects owing to overdosing (13). While clear recommendations exist for TDM of some antimicrobials such as vancomycin or gentamycin (14), evidence on the use of TDM is lacking for a broad variety of other antimicrobial drugs. There are no large studies supporting an evidence-based use of protocolized daptomycin TDM in the ICU (15). Similarly, evidence relating to daptomycin management including validated target concentrations based on large studies is also scarce in non-critically ill patients. While the Area under the concentration curve/Minimal inhibitory concentration ratio (AUC/MIC) is a commonly described efficacy target of daptomycin, practical challenges may complicate its measurement (16, 17). Thus, in order to monitor potential daptomycin toxicity and/or efficacy, measurement of trough or peak concentrations are potential alternatives.

Some previous research using population pharmacokinetic models suggests that, for enhanced drug efficacy, increased dosages might be necessary in critically ill patients and that intravenous daptomycin doses are best scaled by creatinine clearance, with higher doses recommended for patients with hyperrenalism (18). Thus, from a theoretical point of view, the use of daptomycin TDM might have a good rationale and could be associated with clinical benefits in severely ill patients. On the other hand, the drug has been associated with increases in creatine kinase (CK) and skeletal muscle toxicity, particularly in patients undergoing prolonged therapy or in patients with concomitant statin use (19, 20). Samura et al. identified concomitant treatment with statins and antihistamines as risk factors for daptomycin-associated creatine kinase elevation, potentially representing idiosyncratic adverse reactions independent of daptomycin dosage (21). Moreover, eosinophilia and eosinophilic pneumonia are known other side effects that have been reported, underscoring the necessity for close monitoring of blood counts during prolonged daptomycin therapy (22, 23). However, no data obtained from critical care settings exists that assess whether daptomycin TDM in ICU patients might be directly linked to altered dosing regimens based on observed daptomycin levels, treatment durations or whether it might have an impact on the occurrence of side effects. Thus, clinical studies in large cohorts of critically ill patients on daptomycin treatment are necessary to answer existing knowledge gaps.

The aim of this study was the evaluation of baseline daptomycin trough levels in patients undergoing TDM, and how subsequent levels were affected by potential dose adaptations. Further outcomes included daptomycin-free days over 14 days and the occurrence of side effects between patients with and without daptomycin TDM.

## 2 Materials and methods

### 2.1 Study design and patients

This was a retrospective, single-centre study including critically ill, adult (>18 years old) patients hospitalized at the tertiary intensive care unit (ICU) of the University Hospital Zurich (Switzerland) from 01.01.2010 to 19.07.2023. All critically ill patients (i.e., being treated on the ICU) undergoing intravenous daptomycin treatment were assessed for study inclusion. Patients were included irrespective of the medical or surgical diagnosis, including immunosuppressed patients and patients with malignancies. All sources and types (uncomplicated vs. complicated) of infection were eligible for inclusion. Exclusion criteria were age <18 years or documented verbal or written refusal to participate in this study, as assessed by general and ICU-specific consents in our hospital which needed to be signed by patients or their next-of-kin (in case of legal incapacity). The study was conducted according to the principles of the Helsinki Declaration and was approved by the competent local Ethics committee (Cantonal Ethics Commission Zurich, BASEC Number 2023–01379).

### 2.2 Data collection

Data were collected by using our two in-hospital electronic medical records databases, the KISIM (Cistec AG, Zurich, Switzerland) and the Patient Data Management System (PDMS) MetaVision (iMDsoft, Dusseldorf, Germany). Collected data included baseline demographic data (including age, gender and body mass index) and comorbidities (including arterial hypertension, diabetes I/II, chronic liver disease, chronic kidney disease, chronic lung disease, chronic heart failure/coronary artery disease, immunosuppression, history of active malignancy). Furthermore, ICU treatment modalities (e.g., organ support), specific scores (including the Sequential Organ Failure Assessment Score (SOFA) and the Simplified Acute Physiology Score (SAPS II)) and patient outcomes including ICU length of stay and survival were gathered. Daptomycin trough levels (DCtrough), dosages and dosage adaptations over the time of the ICU stay were obtained from the medical records. Potential daptomycin side effects were obtained from written medical records (including diagnosis list) and from daily obtained laboratory values (including creatine kinase (CK) and eosinophil count).

### 2.3 Daptomycin prescribing policy and assessment

In our ICU, intravenous daptomycin is normally administered both for empiric or targeted coverage of Gram-positive microorganisms. The standard dose is 6 mg/kg total body weight once daily intravenously. DCtrough are measured 15 min prior to administration of the new dosage.

Renal dose adjustments are conducted according to the local protocol below an estimated glomerular filtration rate (eGFR) of 30 mL/min/1.73 m2, by prolonging the dose interval to 48 h. During renal replacement therapy (RRT) dose adjustments depend on the modality of dialysis. For continuous renal replacement therapy (CRRT), no dose adjustments are planned. For intermittent hemodialysis (iHD), the dosage interval is 48 h and daptomycin is applied after the hemodialysis session. For sustained low efficiency dialysis (SLED), the normal dosage is applied, but daptomycin is given after SLED.

Although we have the opportunity to measure daptomycin trough levels, we did not have a standardized recommended in-house protocol for the measurement of DCtrough during the study period. Depending on the clinical circumstances such as renal impairment, extracorporeal support or the physicians’ personal preferences, measurements of DCtrough and subsequent dose adjustments are based on the clinical judgment of the treating physicians. Routinely, infectious disease specialists were involved in the treatment of patients. In general, DCtrough of 10 mg/L are targeted in case TDM is used. DCtrough <10 mg/L are considered insufficient based on our in-house laboratory recommendation. Owing to lacking literature, no specific cut-off levels for clearly toxic trough levels exist in our hospital. A pragmatic toxicity threshold was defined as a DCtrough of > 25 mg/L based on results by Bhavnani et al., who reported an increased risk of CK elevation above a DCtrough of >24.3 mg/L (24). In case DCtrough are below the target range, dose adaptations can be prescribed taking pharmacokinetic considerations into account. Potential dose adaptations were routinely discussed interdisciplinary between ICU and infectious disease specialists based on particular circumstances including trough levels, potential side effects or illness severity. After increasing the dose, subsequent daptomycin levels are normally again measured prior to the fifth dose or earlier in case of renal impairment, respectively. Owing to lacking data, there is no specific in-house protocol for laboratory DCtrough assessments in patients undergoing ECMO support. In order to reflect real-world practice, DCtrough were assessed as available from the medical records.

### 2.4 Continuous renal replacement therapy

In our institution, the multiFiltrate Pro CRRT device with AV1000 membranes (mFT, Fresenius Medical Care, Bad Homburg, Germany) is used as a standard machine. The default configuration is continuous veno-venous hemodialysis (CVVHD) with regional citrate anticoagulation (RCA). An initial dialysis dosage of 25 mL/kg/h and a blood flow:dialysate ratio of 1:20 is prescribed, which both can be adapted according to the clinical circumstances such as acid–base disequilibria. In case of citrate accumulation, we usually switch to heparin anticoagulation or continuous veno-venous hemodiafiltration in a predilution mode without citrate administration, depending on the presumed bleeding risk.

### 2.5 Study outcomes

The primary outcome was to assess which initial daptomycin trough levels were achieved in patients using TDM, and how subsequent levels were affected by potential dose adaptations. Further outcomes included whether TDM of daptomycin resulted in more dose adjustments compared to those patients not undergoing TDM, daptomycin-free days alive over 14 days, the occurrence of side effects between patients with and without daptomycin TDM, and an assessment whether daptomycin-related side effects occurred more often in patients with high DCtrough. Specific pre-defined daptomycin-related side effects were elevated creatine kinase (CK) levels and eosinophilia. CK and eosinophil levels were measured daily and defined as elevated if they were above the upper limit of normal of our in-house laboratory (CK > 190 U/L, Eosinophils > 0.7 G/L). Median increases of creatine kinase and eosinophil levels were calculated as baseline values at ICU admission up to maximal values during daptomycin treatment.

### 2.6 Statistical analysis

Data were expressed as absolute numbers and percentages for categorical variables or as median and interquartile ranges IQR (25th–75th percentile) for continuous variables, as appropriate. Group comparisons between patients with and without daptomycin TDM was performed using the Chi-squared test and the Mann–Whitney test, as appropriate.

Statistical analysis was performed through a scripted data pathway using the SPSS (IBM, United States) and JASP (JASP team). A two-sided *p* < 0.05 was considered statistically significant.

## 3 Results

During the inclusion period, 13,952 critically ill patients received antibiotics, of whom 1868 received intravenous daptomycin and were assessed for eligibility. After excluding patients without written consent (n = 405) and those who did not receive daptomycin in the ICU (n = 1,193), a remaining patient count of n = 270 was included in the final analysis. 145 patients had no daptomycin TDM, whereas 125 patients had daptomycin TDM during their ICU stay.

In Table 1, baseline characteristics and laboratory values at ICU admission of the included patients are demonstrated. 28.3% of patients without TDM were female, whereas 32.8% of patients were female in the TDM group. Median age, body mass index, comorbidities and laboratory values were not different between patients with and without TDM (Table 1).

**Table 1:** Baseline characteristics and laboratory values at ICU admission.

ICU admission	No TDM (*n* = 145)	TDM (*n* = 125)	*p*-value
Baseline characteristics			
Age (y)	60 (50–69)	62 (50–70)	0,719
Female	41 (28.3)	41 (32.8)	0,420
Weight (kg)	77 (67–90)	74 (64–89)	0,215
BMI (kg/m2)	25.8 (24.4–28.4)	24,7 (22.5–28.7)	0,194
Obesity (BMI > 30 (kg/m2)	28 (19.3)	22 (17.6)	0.755
Comorbidities	132 (91%)	118 (94.4%)	0.29
Hypertension	64 (44.1)	54 (43.2)	0,903
Diabetes mellitus	39 (26.9)	34 (27.2)	1,000
Chronic liver disease	41 (28.3)	49 (39.2)	0,070
Chronic kidney disease	70 (48.3)	66 (52.8)	0,467
Hemodialysis	11 (7.6)	10 (8)	1,000
Statin use	42 (29)	33 (26.4)	0,684
Laboratory at ICU admission			
eGFR (ml/min)	40 (20–65)	39 (24–60)	0,786
eGFR < 30 ml/min	51 (35.2)	45 (36)	0,899
Albumin (g/l)	26 (21–30)	25 (20–30)	0,345
Creatine kinase (U/l)	95 (45–289)	82 (34–242)	0,262
Eosinophils (G/l)	0,04 (0.01–0.14)	0,04 (0.01–0.13)	0,809

Categorical numbers are reported as count and (percentages). Continuous variables are reported as median and [interquartile range (IQR)]. BMI, Body mass index; eGFR, Estimated glomerular filtration rate; ICU, Intensive care unit; TDM, Therapeutic drug monitoring.

Table 2 demonstrates ICU treatment modalities of patients without and with TDM. SOFA scores at ICU admission were similarly distributed between patients without and with daptomycin TDM (Table 2). Similarly, extracorporeal support modalities (CRRT, iHD, SLED and ECMO) were comparable between patients without and with daptomycin TDM (Table 2). Eosinophilia was more frequent in patients with TDM (Table 2). ICU mortality did not differ between patients without and with TDM, although it was numerically higher in patients with TDM (Table 2). Median ICU length of stay was longer in patients with daptomycin TDM, whereas median daptomycin treatment duration was shorter in patients without TDM (Table 2).

**Table 2:** ICU characteristics and adverse events related to daptomycin.

ICU stay	No TDM (*n* = 145)	TDM (*n* = 125)	*p*-value
ICU characteristics			
SOFA score	10 (8–13)	10 (8–12)	0,45
SAPS II score	45 (32–57)	36 (28–53)	0,031
CRRT	129 (88.9)	111 (88.8)	1,000
iHD	12 (8.3)	13 (10.4)	0,674
SLED	13 (8.9)	10 (8)	0,830
ECMO	22 (15.2)	16 (12.8)	0,603
ICU death	57 (39.3)	56 (44.8)	0,388
In-hospital death	90 (62.1)	91 (72.8)	0,070
ICU length of stay (days)	15 (7–28)	18 (10–32)	0,027
Adverse events			
High CK levels	30 (20.7)	28 (22.4)	0,334
Eosinophilia	16 (11)	23 (18.4)	0,047
High CK levels + Eosinophilia	9 (6.2)	7 (5.6)	0,813

Categorical numbers are reported as count and (percentages). Continuous variables are reported as median and (interquartile range (IQR)). CK, Creatine kinase; CRRT, Continuous renal replacement therapy; ECMO, Extracorporeal membrane oxygenation; ICU, Intensive care unit; iHD, Intermittend hemodialysis; SAPS, Simplified acute physiology score; SLED, Sustained low efficiency dialysis; SOFA, Sequential organ failure assessment.

In patients without TDM, baseline daptomycin dosing was 6 mg/kg (IQR 5.6–6.0) and in patients with TDM 6 mg/kg (IQR 5.6–6.4) (*p* = 0.63). In patients with TDM, median DCtrough was 9 mg/L (IQR 5.8–16.4 mg/L). Median daptomycin peak levels were 29. 8 mg/L (IQR 14.8 – 46 mg/L). In total, 172 DCtrough (on average 1.4 DCtrough per patient in the TDM group) and 100 peak levels (on average 0.8 levels per patient in the TDM group) were obtained. The distribution of DCtrough (n = 172 values) is shown in Supplementary Figure 1. Because treatment efficacy may depend largely on achieving sufficiently high daptomycin peak concentrations and values up to 43 mg/L have been suggested as effective in clinical practice (25, 26), our observed median peak concentration of 29.8 mg/L may have contributed to suboptimal treatment responses in a subset of our patient cohort, which is further supported by the lower bound of the daptomycin peak IQR, which was only 14.8 mg/L. Supplementary Figure 2 shows daptomycin trough and peak levels stratified for patients on CRRT, SLED, iHD and ECMO.

In the TDM group, in 108 out of 125 patients a DCtrough was available. In 17 patients only peak levels were available. Initial DCtrough were <10 mg/L in 62 patients (54.4%), within the normal range in 41 patients (38%) and > 25 mg/L in only 5 patients (4.6%) (Table 3; Supplementary Figure 3). In patients with initial too low DCtrough, dosage was increased in 14 patients (22.6%) and left unchanged in 42 patients (67.7%) (Supplementary Figure 3). Only 15 out of 62 patients (24.1%) with an initial too low DCtrough subsequently reached normal DCtrough. Median DCtrough after dosage increases was 7 mg/L (IQR 4.8–13.6 mg/L).

**Table 3:** Initial daptomycin trough levels and dose adaptations in 108 out of 125 patients with TDM.

Initial daptomycin trough level	Patients (%)
< 10 mg/l	62 patients (54.4%)
10-25 mg/l	41 patients (38%)
> 25 mg/l	5 patients (4.6%)
Subsequent dose adaptations in patients with low daptomycin trough levels	
Increased	14 patients (22.6%)
Left unchanged	42 patients (67.7%)
Decreased	6 patients (9.7%)

Numbers reported in count and (percentages). In 17 patients of the TDM group (*n* = 125), only daptomycin peak levels were available and no trough levels.

**Figure 1:**
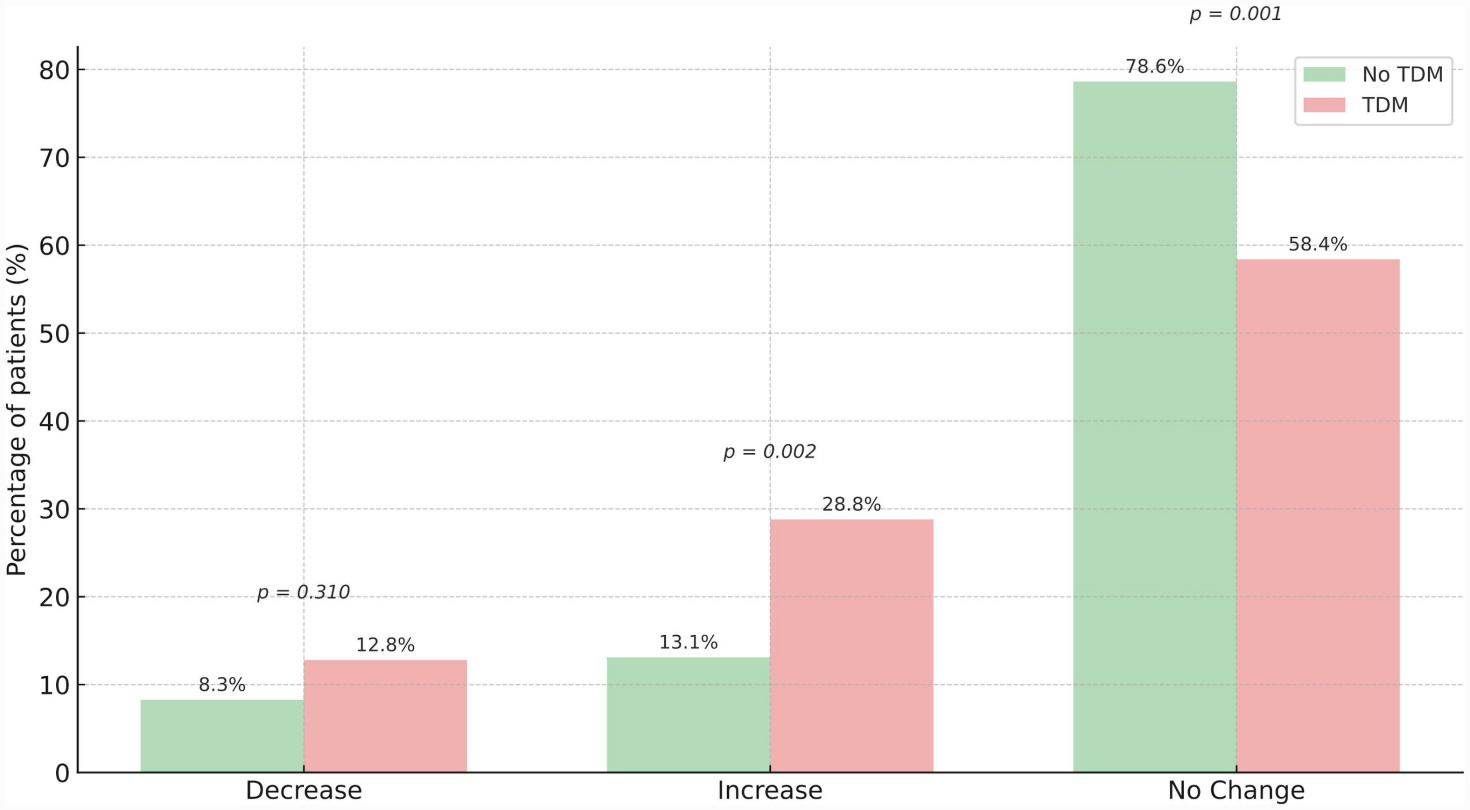
Daptomycin dose adjustments: no TDM vs. TDM. Daptomycin dose adjustments (decrease, increase, no change) for patients without (green color) and with (red color) daptomycin therapeutic drug monitoring (TDM).

Figure 1 demonstrates dose adaptations (increases, decreases, no change) for patients without and with TDM. Dosage decreases were not statistically different between patients without and with TDM (8.3% vs. 12.8%, *p* = 0.31). In patients with TDM, daptomycin dosages were significantly more often increased compared to patients without TDM (28.8% vs. 13.1%, *p* = 0.002). Similarly, daptomycin doses were not changed more often in patients without TDM compared to patients with TDM (78.6% vs. 58.4%, *p* = 0.001; Figure 1).

**Figure 2:**
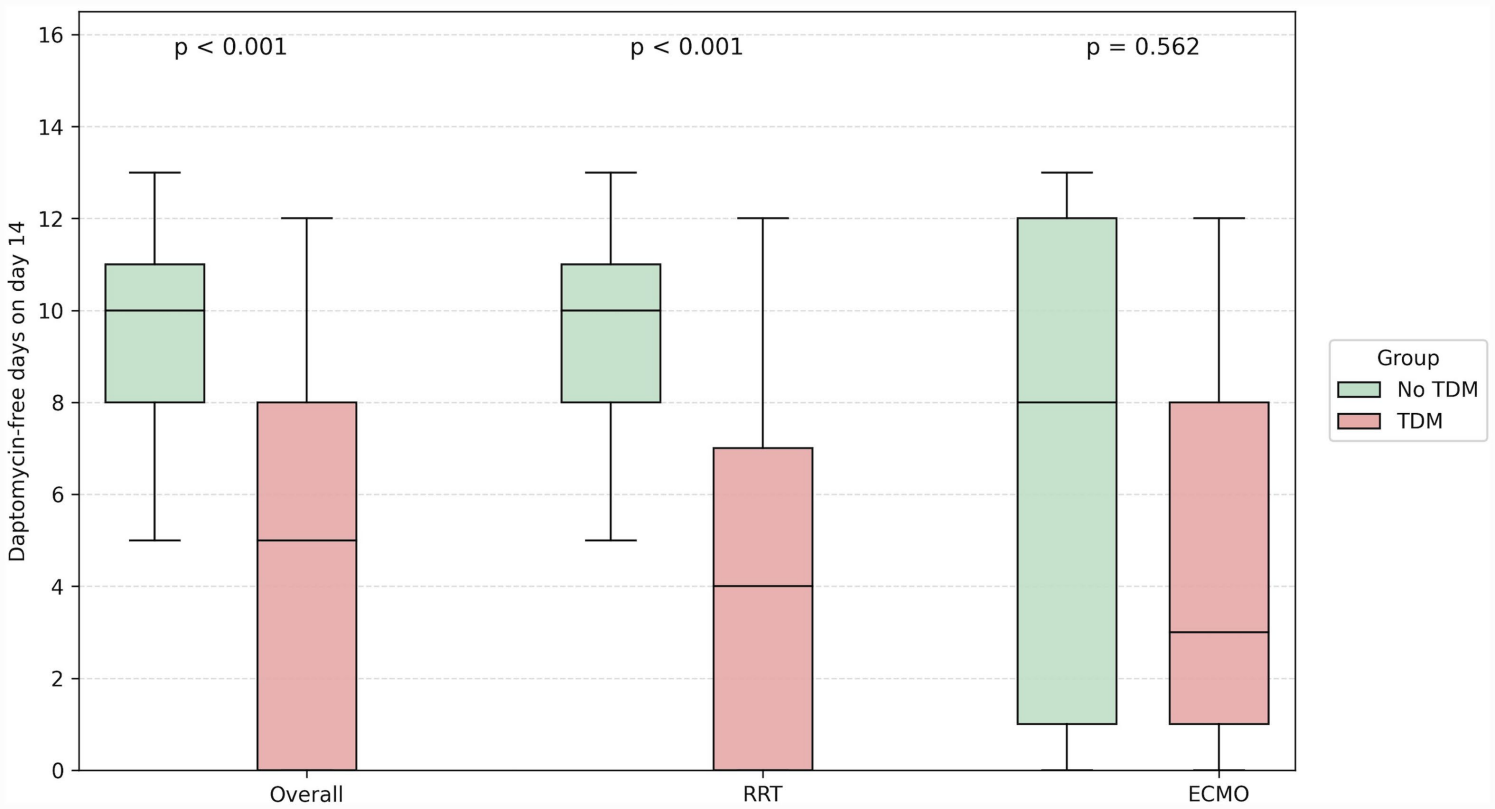
Distribution of daptomycin-free days. Daptomycin-free days alive for patients without (green) and with (red) therapeutic drug monitoring (TDM). For both groups, stratifications occurred for patients overall, for patients on renal replacement therapy (RRT), and for patient on extracorporeal membrane oxygenation (ECMO).

In Figure 2, daptomycin-free days over 14 days are demonstrated overall, and stratified according to patients undergoing RRT and ECMO therapy. Overall, daptomycin-free days were shorter in patients undergoing TDM compared to patients without TDM [5 days (IQR 0–8 days) vs. 10 days (IQR 8–11 days), *p* < 0.001]. Similar findings were obtained for patients with TDM and RRT [4 days (IQR 0–8 days) vs. 10 days (IQR 8–11 days), *p* < 0.001]. Daptomycin-free days were numerically shorter for patients with TDM and ECMO support, however without statistical significance [3 days (IQR 1–8 days) vs. 8 days (IQR 1–12 days), *p* = 0.56].

**Figure 3:**
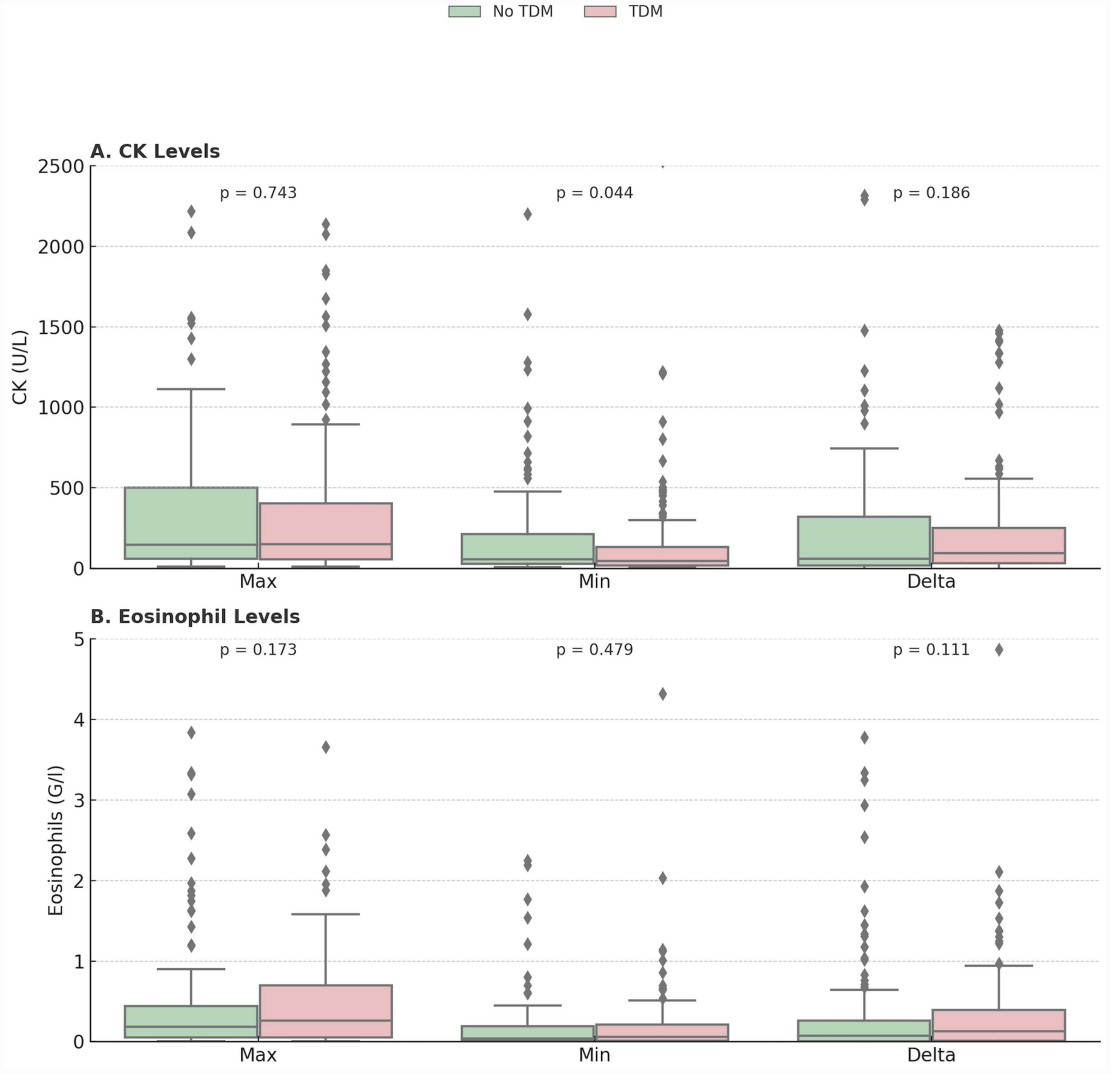
CK and eosinophil levels by TDM status. Maximal and minimal creatine kinase (Panel A) and eosinophil levels (Panel B) during daptomycin treatment for patients without (green color) and with (red color) therapeutic drug monitoring. Median increases of creatine kinase (Panel A) and eosinophil levels (Panel B) during daptomycin treatment for patients without (green color) and with (red color) therapeutic drug monitoring.

Figure 3 demonstrates median increases of creatine kinase and eosinophil for patients without and with TDM. Maximal and minimal creatine kinase and eosinophil levels during daptomycin treatment were not different between groups (Figure 3).

The TDM group exhibited a numerically higher, but not statistically significant, median increase in CK levels (TDM 93 U/L vs. no TDM 57 U/L, *p* = 0.186) and eosinophil counts (TDM 0.13 G/L vs. no TDM 0.07 G/L, *p* = 0.111).

Overall, in patients with high DCtrough (> 25 mg/L) measured anytime during their daptomycin treatment (*n* = 41), elevated CK levels occurred in 12 patients (29%) and eosinophilia in 2 patients (5%), with subsequent dosage reductions in 4 patients (28.6%). In patients without daptomycin TDM (*n* = 145) and observed increased CK levels (*n* = 30) and/or eosinophil counts (*n* = 11), daptomycin dosage was only reduced in 5 patients. Increased CK levels and eosinophilia also occurred in patients without elevated DCtrough (*n* = 39). Daptomycin dosage was subsequently reduced in 5 patients (12.8%). No further adverse events related to daptomycin (e.g., allergic shock) were documented in the medical records for all patients.

## 4 Discussion

This study aimed to assess what daptomycin trough levels were achieved in patients using TDM, and how subsequent levels were affected by dose adaptations. Further outcomes included whether TDM of daptomycin resulted in dose adjustments compared to those patients not undergoing TDM, daptomycin-free days over 14 days, the occurrence of side effects such as elevated CK and eosinophilia between patients with and without daptomycin TDM. Our results demonstrate that DCtrough are often insufficient in critically ill patients, and DCtrough frequently do not increase despite dose adaptations by clinicians. Interestingly, dose increases were uncommonly performed despite low DCtrough. Moreover, patients undergoing TDM had significantly more frequent dose increases of daptomycin compared to their counterparts without TDM. Patients with TDM also experienced fewer daptomycin-free days. Increases in CK and eosinophil count did not differ significantly between groups, while eosinophilia was slightly more common in patients with TDM.

An overall clinically relevant finding of our study was the commonly low observed DCtrough. It has been characterized that, particularly in ICU patients often supported by RRT and/or ECMO therapy, currently recommended standard dosage regimens can be insufficient and higher than standard doses might be necessary (3, 27). In this study, we however observed frequently lacking effects on DCtrough despite dose increases in cases where it was performed. More frequent dose increases in the TDM group could mirror attempts of clinicians to reach adequate target trough levels, especially in our rather sick cohort with frequent extracorporeal therapies such as RRT and ECMO. This raises the relevant question to what extent sufficient DCtrough can be reached without administering toxic daptomycin doses, a question that needs to be addressed in future prospective trials including similar critically ill patients. Despite lacking clear international guidelines, TDM can provide relevant pharmacokinetic information particularly in ICU patients with highly variable pharmacokinetics. Our finding also highlights the necessity of observing measured DCtrough, as clinicians often did not draw consequences and left daptomycin dosage unchanged despite observed low DCtrough. We further observed that toxic daptomycin levels rarely occurred and that dose decreases were comparable between patients with and without daptomycin TDM. This observation can be explained by the fact that overall median trough levels in our cohort were around the target level of 10 mg/L, making dose decreases unnecessary. In line with our finding, it has been reported that adequate daptomycin exposure can be difficult to achieve in critically ill patients (1). Nevertheless, our median DCtrough were higher than previously reported insufficient levels, where a higher risk of poor clinical outcomes was observed (28). Albeit DCtrough analyses play important roles in the evaluation of the safety profile of daptomycin, it has been described that treatment efficacy mainly relates to daptomycin peak concentrations (25). This highlights the clinical need to also consider measuring daptomycin peak concentrations especially in ICU settings, where optimal antimicrobial effects are crucial for patient management.

In general, it also needs to be mentioned, that especially in the ICU setting, several clinical parameters have impacts on daptomycin exposure and should be considered by ICU clinicians when daptomycin is administered. For example, one study assessed daptomycin exposure after a single dose in morbidly obese patients and found that maximum plasma concentration and area under the concentration-time curve was 60% higher in obese patients, highlighting the need to take body weight into account in clinics and in further studies (29). Future studies should also assess the impact of renal function on daptomycin pharmacokinetics and dosing regimens, especially in the early course of antimicrobial treatment (30–32). Moreover, further distinct pathophysiological alterations in critically ill patients such as increased or decreased cardiac output, hepatic dysfunction, altered protein binding, capillary leakage, the use of extracorporeal devices or effects of massive fluid resuscitation could affect drug pharmacokinetics and volume of distribution of daptomycin, underscoring the need for further prospective investigations (30).

The reduction in daptomycin-free days in the TDM group is most likely related to longer overall daptomycin treatment durations. Patients receiving TDM also had longer ICU stays, a factor that may have increased the likelihood of TDM initiation and reflects confounding by indication rather than a causal effect of TDM itself. Clinicians might also have prolonged daptomycin therapy in response to low trough concentrations. These complex relationships cannot be disentangled within the observational design of the present study. Although ICU mortality was numerically higher in the TDM group, the study was not powered to detect mortality differences, and this finding may be more likely attributable to other factors among patients undergoing TDM.

While TDM was associated with a higher frequency of dose adjustments, this did not consistently translate into higher daptomycin trough concentrations. Thus, TDM could be interpreted as a tool supporting individualized dose adjustments rather than as evidence of systematically higher or more targeted exposure. TDM was not associated with a reduction in adverse effects events related to daptomycin such as increases in CK. In contrast, there was a potential signal toward a higher frequency of eosinophilia in patients with TDM. This finding could be linked to the relatively low incidence of these events in our cohort, or to the fact that daptomycin peak levels were not substantially elevated in our cohort, irrespective of extracorporeal support devices. Indeed, our observed daptomycin peak levels were only moderately higher than previously reported toxic trough levels (15, 24), which is again in line with previous data suggesting that adequate daptomycin exposure might be difficult to achieve in ICU patients (1, 33). Moreover, it has been described that especially severe daptomycin-related complications such as eosinophilic pneumonia (which did not occur in our cohort) frequently develop after daptomycin treatment durations greater than 14 days (34), which is longer than the median treatment exposure in our own patients. The absolute rate of eosinophilia was slightly higher in patients undergoing TDM. This finding might relate to longer daptomycin treatment durations in patients with TDM or to the fact that clinicians might measure daptomycin levels in case eosinophilia occurs. From our retrospective design, we cannot conclude if this is a causal association. Interestingly, we infrequently observed elevated CK levels and eosinophilia in patients with too high DCtrough over their ICU stay. In light of the rarity of eosinophilic pneumonia, we consider daily measurement of eosinophil count a reasonable approach for early detection of potential drug-associated side effects by clinicians.

Conversely, in patients without daptomycin TDM and increased CK levels and/or eosinophil counts, daptomycin dosage was only reduced in 5 patients, underscoring the need for clinicians to closely monitor potential side effects and to carefully evaluate potential changes to another antibiotic. Importantly, no cases of allergic reactions were documented in our study, also in patients without TDM. Regarding the association between high-dose daptomycin regimens and subsequent adverse events, available literature suggests that high-dose therapy is usually associated with drug overexposure, but the risk of toxicity linked to these regimens could be reduced, particularly in case of shorter treatment durations and when TDM is used (35, 36). In a study of 77 patients undergoing daptomycin treatment, Angelini et al. found that CK during therapy increased in only 6 patients and eosinophil count > 500/ul occurred in less than 10% of patients (36). Nevertheless, our data show that specific drug-related side effects can also occur in the presence of presumed normal daptomycin levels. Importantly, clinicians should be aware of potential risk factors leading to daptomycin overexposure such as renal function, BMI and co-medications (37).

Our findings suggest that daptomycin TDM in the ICU setting may be associated with changes in dosing behavior among ICU physicians, while we did not observe clear evidence of an association with patient outcomes, including safety surrogates such as muscle or eosinophilic toxicity. This would need to be assessed in a controlled, protocolized trough level assessment and subsequent intervention. Our results may indicate a potentially greater role of TDM in monitoring therapeutic exposure rather than toxicity, especially in clinical contexts characterized by high pharmacokinetic variability (1, 3). Nevertheless, the clinical relevance of this observation remains uncertain, and no internationally accepted recommendations for daptomycin TDM in critically ill patients are currently available. Future prospective studies are needed to define optimal daptomycin TDM strategies in critically ill patients. Additionally, randomized controlled trials could help to determine whether protocolized TDM leads to improved clinical outcomes such as resolution of infection, reduced antimicrobial resistance, or safety benefits such as a lower rate of side effects. Future trials could also directly correlate daptomycin concentrations with antimicrobial killing, which so far only has been assessed in *in vitro* or animal studies, where area under the concentration curve (AUC)/minimal inhibitory concentration (MIC) ratio and peak concentrations (Cmax)/MIC ratio were the main factors related to bactericidal effects (38, 39). In addition, future alternative dosing and monitoring regimens of daptomycin are of high interest and should be validated prospectively (26, 40, 41). Olney et al. investigated the use of fixed daptomycin dosing and found improved efficacy-to-toxicity ratios and lower costs compared to weight-based doses (40). Angelini et al. analyzed an alternative TDM approach suggesting daptomycin daily AUC estimations with a single sample collected 7–11 h after dose initiation (26). In contrast to DCtrough measurements, the authors thereby suggested concentrations of 30 mg/L to 43 mg/L to be adequate starting points facilitating precision dosing (26). Other regimens also could include mid-interval sampling as a practical approach compared to peak and trough sampling (41). Giuliano et al. found that a mid-interval daptomycin sampling strategy offered more consistent AUC estimation (41).

Strengths of our study include a relatively large patient cohort and real-world data reflecting current clinical practice over a long observational period including patients with extracorporeal support. The inclusion of clinically relevant endpoints such as side effects enhances the clinical and practical value of our results. The study also has to account for some limitations. It is limited by its retrospective, single-center design, which could introduce potential biases and might limit generalizability. Moreover, daptomycin levels were not measured in a protocolized manner and overall daptomycin treatment durations might have been too short for the development of specific daptomycin-related side effects. Due to the retrospective design, reasons for dose adaptations are difficult to characterize. Furthermore, other unmeasured confounders such as infection type, illness severity or ICU length of stay might have affected our findings. The absence of standardized protocols for TDM implementation and associated dose adjustments further might have introduced variability in clinical decisions. Nevertheless, our study reflects real-life practice and mirrors the absence of clear guideline-based recommendations. Finally, our study mainly analyzed daptomycin trough levels based on available measurements and reflecting our institution’s approach to daptomycin TDM, whereas structured measurements of peak levels might yield additional benefits regarding treatment efficacy.

## 5 Conclusion

Daptomycin levels are commonly low in critically ill patients and often do not increase after dose adjustments. TDM was associated with more frequent dose escalations and fewer daptomycin-free days, but did not significantly reduce the incidence of adverse events in a large cohort of critically ill patients. The findings highlight the role of TDM in guiding individualized dosing, although the clinical impact on safety and further patient outcomes warrants further evaluation in prospective studies with structured protocols for daptomycin level assessment and subsequent dosing adaptations.

## Data Availability

The raw data supporting the conclusions of this article will be made available by the authors, without undue reservation.

## References

[1] D’Avolio A Pensi D Baietto L Pacini G Di Perri G De Rosa FG. Daptomycin pharmacokinetics and pharmacodynamics in septic and critically ill patients. Drugs. (2016) 76:1161–74. doi: 10.1007/s40265-016-0610-3, 27412121

[2] Alder J. The use of daptomycin for *Staphylococcus aureus* infections in critical care medicine. Crit Care Clin. (2008) 24:349–63. doi: 10.1016/j.ccc.2007.12.010, 18361950

[3] Zhang LC Li QY Zhang YQ Shan TC Li Y Li YH . Population pharmacokinetics of daptomycin in critically ill patients receiving extracorporeal membrane oxygenation. J Antimicrob Chemother. (2024) 79:1697–705. doi: 10.1093/jac/dkae171, 38814793

[4] Zhang Y Hu H Zhang Q Ou Q Zhou H Sha T . Effects of ex vivo extracorporeal membrane oxygenation circuits on sequestration of antimicrobial agents. Front Med. (2021) 8:748769. doi: 10.3389/fmed.2021.748769, 34926498 PMC8671752

[5] Shekar K Abdul-Aziz MH Cheng V Burrows F Buscher H Cho YJ . Antimicrobial exposures in critically ill patients receiving extracorporeal membrane oxygenation. Am J Respir Crit Care Med. (2023) 207:704–20. doi: 10.1164/rccm.202207-1393OC, 36215036

[6] Xie F Li S Cheng Z. Population pharmacokinetics and dosing considerations of daptomycin in critically ill patients undergoing continuous renal replacement therapy. J Antimicrob Chemother. (2020) 75:1559–66. doi: 10.1093/jac/dkaa028, 32083673

[7] Hoff BM Maker JH Dager WE Heintz BH. Antibiotic dosing for critically ill adult patients receiving intermittent hemodialysis, prolonged intermittent renal replacement therapy, and continuous renal replacement therapy: an update. Ann Pharmacother. (2020) 54:43–55. doi: 10.1177/1060028019865873, 31342772

[8] Morales Castro D Dresser L Granton J Fan E. Pharmacokinetic alterations associated with critical illness. Clin Pharmacokinet. (2023) 62:209–20. doi: 10.1007/s40262-023-01213-x, 36732476 PMC9894673

[9] Patel JS Kooda K Igneri LA. A narrative review of the impact of extracorporeal membrane oxygenation on the pharmacokinetics and pharmacodynamics of critical care therapies. Ann Pharmacother. (2023) 57:706–26. doi: 10.1177/10600280221126438, 36250355

[10] Kim M Mahmood M Estes LL Wilson JW Martin NJ Marcus JE . A narrative review on antimicrobial dosing in adult critically ill patients on extracorporeal membrane oxygenation. Crit Care. (2024) 28:326. doi: 10.1186/s13054-024-05101-z, 39367501 PMC11453026

[11] Camen G Wendel-Garcia PD Erlebach R Müller M John C Buhlmann A . Teicoplanin pharmacokinetics in critically ill patients on extracorporeal organ support: a retrospective analysis. Intensive Care Med Exp. (2025) 13:22. doi: 10.1186/s40635-025-00729-9, 39982576 PMC11845331

[12] Grégoire N Marchand S Ferrandière M Lasocki S Seguin P Vourc'h M . Population pharmacokinetics of daptomycin in critically ill patients with various degrees of renal impairment. J Antimicrob Chemother. (2019) 74:117–25. doi: 10.1093/jac/dky374, 30295740

[13] Takahashi N Kondo Y Kubo K Egi M Kano KI Ohshima Y . Efficacy of therapeutic drug monitoring-based antibiotic regimen in critically ill patients: a systematic review and meta-analysis of randomized controlled trials. J Intensive Care. (2023) 11:48. doi: 10.1186/s40560-023-00699-8, 37936203 PMC10631080

[14] Rybak M Lomaestro B Rotschafer JC Moellering R Craig W Billeter M . Therapeutic monitoring of vancomycin in adult patients: a consensus review of the American Society of Health-System Pharmacists, the Infectious Diseases Society of America, and the Society of Infectious Diseases Pharmacists. Am J Health Syst Pharm. (2009) 66:82–98. doi: 10.2146/ajhp080434, 19106348

[15] Abdul-Aziz MH Alffenaar JC Bassetti M Bracht H Dimopoulos G Marriott D . Antimicrobial therapeutic drug monitoring in critically ill adult patients: a position paper. Intensive Care Med. (2020) 46:1127–53. doi: 10.1007/s00134-020-06050-1, 32383061 PMC7223855

[16] European Committee on Antimicrobial Susceptibility Testing (EUCAST) Steering Committee. EUCAST technical note on daptomycin. Clin Microbiol Infect. (2006) 12:599–601. doi: 10.1111/j.1469-0691.2006.01455.x 16700717

[17] Louie A Kaw P Liu W Jumbe N Miller MH Drusano GL. Pharmacodynamics of daptomycin in a murine thigh model of *Staphylococcus aureus* infection. Antimicrob Agents Chemother. (2001) 45:845–51. doi: 10.1128/AAC.45.3.845-851.2001, 11181370 PMC90383

[18] Wu J Zheng X Zhang L Wang J Lv Y Xi Y . Population pharmacokinetics of intravenous daptomycin in critically ill patients: implications for selection of dosage regimens. Front Pharmacol. (2024) 15:1378872. doi: 10.3389/fphar.2024.1378872, 38756382 PMC11096781

[19] Dare RK Tewell C Harris B Wright PW Van Driest SL Farber-Eger E . Effect of statin coadministration on the risk of daptomycin-associated myopathy. Clin Infect Dis. (2018) 67:29668884:1356–63. doi: 10.1093/cid/ciy287 PMC6186852 29668884

[20] Echevarria K Datta P Cadena J Lewis JS. Severe myopathy and possible hepatotoxicity related to daptomycin. J Antimicrob Chemother. (2005) 55:599–600. doi: 10.1093/jac/dki058, 15743894

[21] Samura M Hirose N Kurata T Takada K Nagumo F Koshioka S . Identification of risk factors for daptomycin-associated creatine phosphokinase elevation and development of a risk prediction model for incidence probability. Open Forum Infect Dis. (2021) 8:ofab568. doi: 10.1093/ofid/ofab568, 34888403 PMC8651170

[22] Bartal C Sagy I Barski L. Drug-induced eosinophilic pneumonia: a review of 196 case reports. Medicine (Baltimore). (2018) 97:e9688. doi: 10.1097/MD.0000000000009688, 29369189 PMC5794373

[23] Pham TT Garreau R Craighero F Cottin V Said BB Goutelle S . Seventeen cases of daptomycin-induced eosinophilic pneumonia in a cohort of patients treated for bone and joint infections: proposal for a new algorithm. Open Forum Infect Dis. (2022) 9:ofac577. doi: 10.1093/ofid/ofac577, 36447615 PMC9697587

[24] Bhavnani SM Rubino CM Ambrose PG Drusano GL. Daptomycin exposure and the probability of elevations in the creatine phosphokinase level: data from a randomized trial of patients with bacteremia and endocarditis. Clin Infect Dis. (2010) 50:1568–74. doi: 10.1086/652767, 20462352

[25] Falcone M Russo A Venditti M Novelli A Pai MP. Considerations for higher doses of daptomycin in critically ill patients with methicillin-resistant *Staphylococcus aureus* bacteremia. Clin Infect Dis. (2013) 57:1568–76. doi: 10.1093/cid/cit582, 24046298

[26] Angelini J Liu S Giuliano S Flammini S Martini L Tascini C . Revolutionizing Daptomycin dosing: a single 7-11-hour sample for pragmatic application. Clin Infect Dis. (2024) 79:596–9. doi: 10.1093/cid/ciae178, 38552199

[27] Reiber C Senn O Müller D Kullak-Ublick GA Corti N. Therapeutic drug monitoring of daptomycin: a retrospective monocentric analysis. Ther Drug Monit. (2015) 37:634–40. doi: 10.1097/FTD.0000000000000196, 26384039

[28] Galar A Muñoz P Valerio M Cercenado E García-González X Burillo A . Current use of daptomycin and systematic therapeutic drug monitoring: clinical experience in a tertiary care institution. Int J Antimicrob Agents. (2019) 53:40–8. doi: 10.1016/j.ijantimicag.2018.09.015, 30243587

[29] Pai MP Norenberg JP Anderson T Goade DW Rodvold KA Telepak RA . Influence of morbid obesity on the single-dose pharmacokinetics of daptomycin. Antimicrob Agents Chemother. (2007) 51:2741–7. doi: 10.1128/AAC.00059-07, 17548489 PMC1932544

[30] Roger C Koulenti D Novy E Roberts J Dahyot-Fizelier C. Optimization of antimicrobial therapy in critically ill patients, what clinicians and searchers must know. Anaesth Crit Care Pain Med. (2025) 44:101561. doi: 10.1016/j.accpm.2025.101561, 40441484

[31] Angelini J Giuliano S Lanini S Ferin S Martini L Cossettini S . In reply to the letter to editor regarding 'renal function and its impact on the concentration of ceftazidime-avibactam: a cross-sectional study'. Int J Antimicrob Agents. (2025) 65:107480. doi: 10.1016/j.ijantimicag.2025.107480, 40057139

[32] Crass RL Rodvold KA Mueller BA Pai MP. Renal dosing of antibiotics: are we jumping the gun? Clin Infect Dis. (2019) 68:1596–602. doi: 10.1093/cid/ciy790, 30219824

[33] Soraluce A Asín-Prieto E Rodríguez-Gascón A Barrasa H Maynar J Carcelero E . Population pharmacokinetics of daptomycin in critically ill patients. Int J Antimicrob Agents. (2018) 52:158–65. doi: 10.1016/j.ijantimicag.2018.03.008, 29572042

[34] Uppal P LaPlante KL Gaitanis MM Jankowich MD Ward KE. Daptomycin-induced eosinophilic pneumonia - a systematic review. Antimicrob Resist Infect Control. (2016) 5:55. doi: 10.1186/s13756-016-0158-8, 27999664 PMC5153904

[35] Garreau R Pham TT Bourguignon L Millet A Parant F Bussy D . Daptomycin exposure as a risk factor for Daptomycin-induced eosinophilic pneumonia and muscular toxicity. Clin Infect Dis. (2023) 77:1372–80. doi: 10.1093/cid/ciad386, 37467019

[36] Angelini J Giuliano S Russiani F Lo Re F Flammini S Cadeo B . PK/PD analysis of high-dose daptomycin use in the treatment of bone and joint infections: data from a real-world setting. Microorganisms. (2025) 13:304. doi: 10.3390/microorganisms13020304, 40005671 PMC11858051

[37] Vellat C Garreau R Millet A Piron C Bourguignon L Roux S . Risk factors of daptomycin overexposure: a case-control study. Antimicrob Agents Chemother. (2025) 69:e0113925. doi: 10.1128/aac.01139-25, 41201893 PMC12691659

[38] Safdar N Andes D Craig WA. In vivo pharmacodynamic activity of daptomycin. Antimicrob Agents Chemother. (2004) 48:63–8. doi: 10.1128/AAC.48.1.63-68.2004, 14693519 PMC310158

[39] Hanberger H Nilsson LE Maller R Isaksson B. Pharmacodynamics of daptomycin and vancomycin on *Enterococcus faecalis* and *Staphylococcus aureus* demonstrated by studies of initial killing and postantibiotic effect and influence of Ca2+ and albumin on these drugs. Antimicrob Agents Chemother. (1991) 35:1710–6. doi: 10.1128/AAC.35.9.1710, 1659305 PMC245255

[40] Olney KB Pai MP Thomas JK Burgess DR Olney WJ Bruning RA . Fixed dose daptomycin: an opportunity for pharmacokinetic/pharmacodynamic optimization in *Staphylococcus aureus* infections. Pharmacotherapy. (2024) 44:615–22. doi: 10.1002/phar.4602, 39078247

[41] Giuliano S Pai MP Angelini J Flammini S Martini L Baraldo M . Precision Daptomycin dosing: comparison of 3-, 2-, 1-, and 0-concentration sample strategies. Pharmacotherapy. (2025) 45:540–6. doi: 10.1002/phar.70045, 40772492 PMC12424523

