# Supplementary material for: Therapeutic drug monitoring of daptomycin in a critically ill patient cohort
Source: Front Med (Lausanne). 2026 Feb 11;13:1732645. doi: 10.3389/fmed.2026.1732645 (PMC12932621; doi:10.3389/fmed.2026.1732645)
Supplement: Supplementary file 1 [file Data_Sheet_1.docx]

Supplementary Material

# Supplemental Figure 1


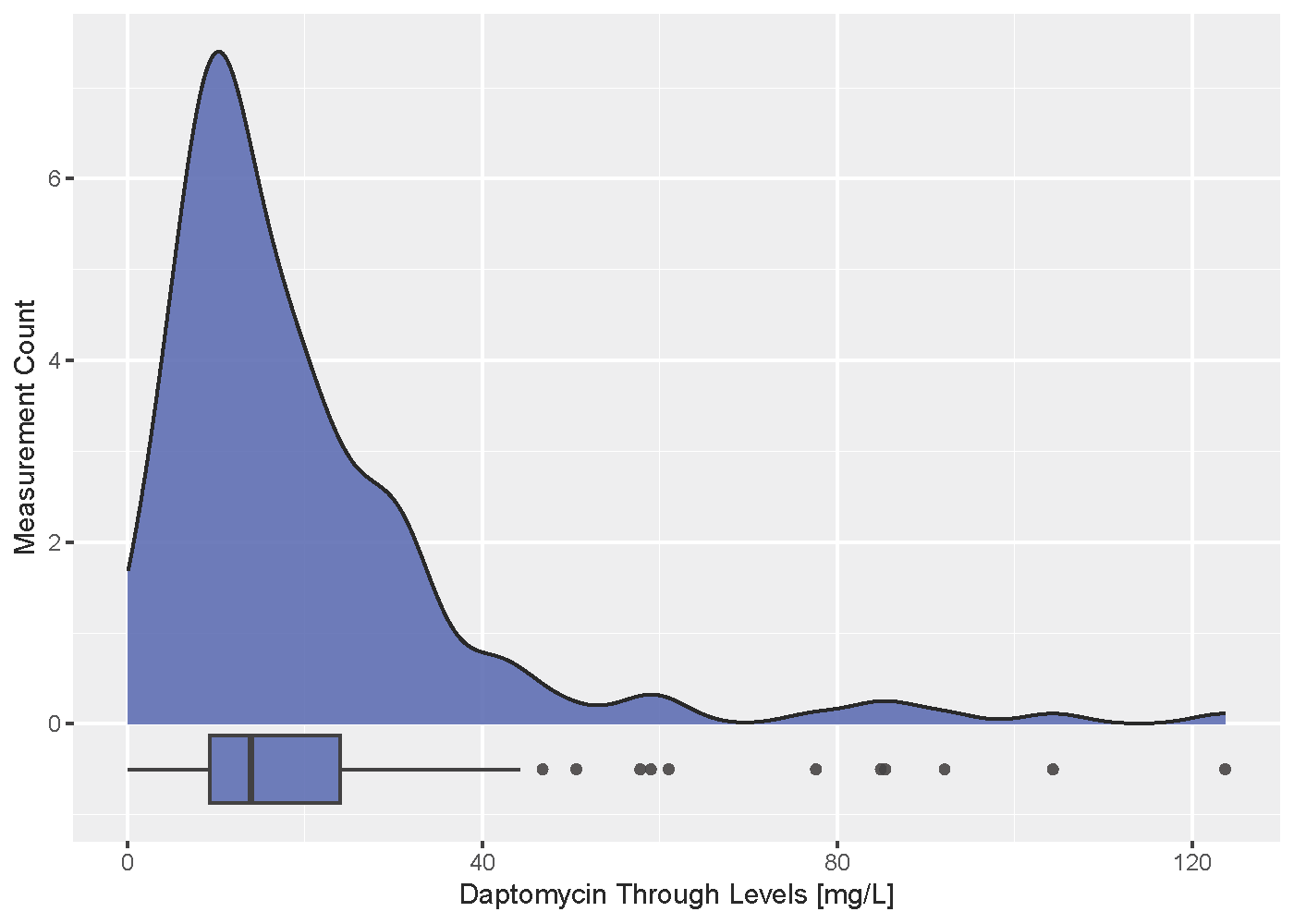


**Supplemental Figure 1.** Distribution of daptomycin trough levels (n=172) in mg/l.

# Supplemental Figure 2


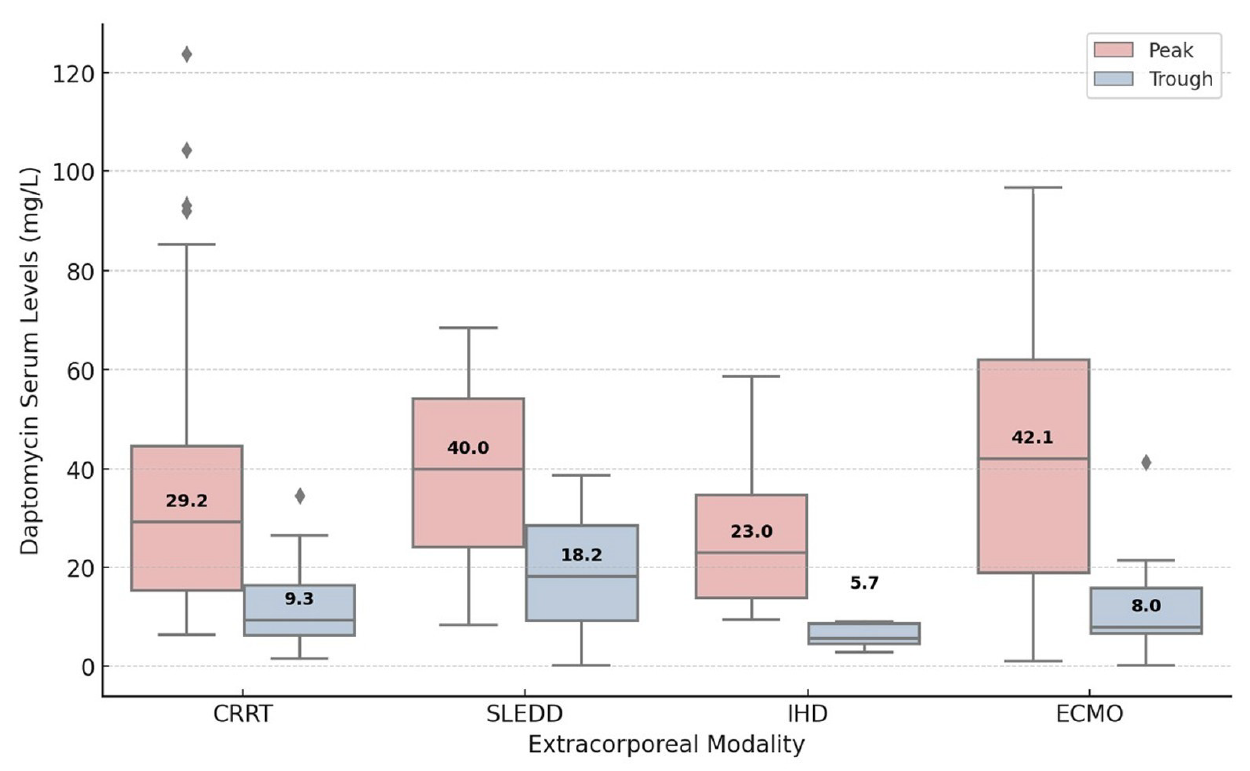


**Supplemental Figure 2.** Difference between daptomycin trough (blue) and peak levels (red) according to extracorporeal treatment modality.

# Supplemental Figure 3
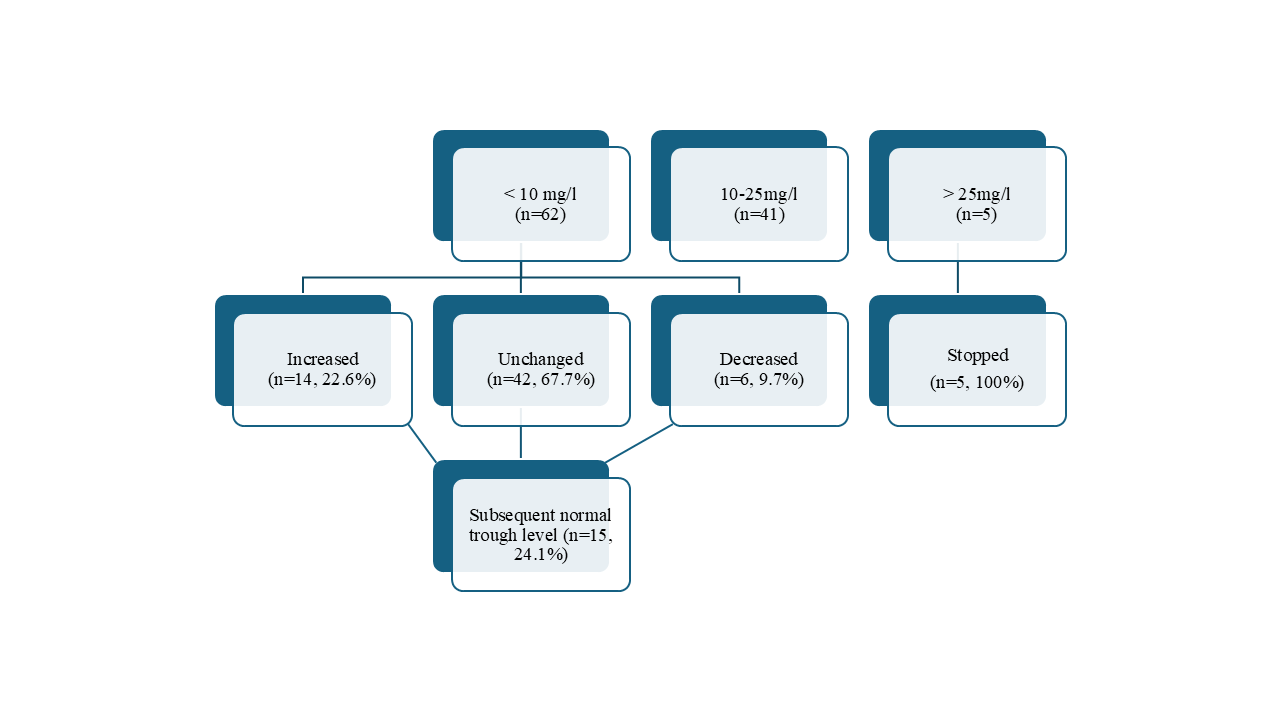


**Supplemental Figure 3.** Initial daptomycin trough levels and subsequent dose adaptations in 108 out of 125 patients with TDM. In 17 patients of the TDM group (n=125), only daptomycin peak levels were available and no trough levels.
